# Bariatric Surgery during COVID-19 Pandemic from Patients’ Point of View—The Results of a National Survey

**DOI:** 10.3390/jcm9061697

**Published:** 2020-06-02

**Authors:** Maciej Walędziak, Anna Różańska-Walędziak, Michał Pędziwiatr, Jacek Szeliga, Monika Proczko-Stepaniak, Michał Wysocki, Tomasz Stefura, Piotr Major

**Affiliations:** 1 Department of General, Oncological, Metabolic and Thoracic Surgery, Military Institute of Medicine, 04-141 Warsaw, Poland; maciej.waledziak@gmail.com; 2 2nd Department of Obstetrics and Gynecology, Medical University of Warsaw, 00-315 Warsaw, Poland; aniaroza@tlen.pl; 3 2nd Department of General Surgery, Jagiellonian University Medical College, 31-008 Kraków, Poland; mpedziwiatr@googlemail.com (M.P.); m.wysocki@doctoral.uj.edu.pl (M.W.); tomasz.stefura@gmail.com (T.S.); 4 Department of General, Gastroenterological, and Oncological Surgery, Collegium Medicum Nicolaus Copernicus University, 85-067 Torun, Poland; jacky2@wp.pl; 5 Department of General, Endocrine and Transplant Surgery, Medical University of Gdansk, 80-210 Gdansk, Poland; monika.proczko-stepaniak@gumed.edu.pl

**Keywords:** bariatric surgery, COVID

## Abstract

Introduction: The aim of the study was to investigate the impact of the COVID-19 pandemic on bariatric care from the patients’ point of view. The COVID-19 pandemic has perturbed the functioning of healthcare systems around the world and led to changes in elective surgical care, with bariatric procedures being postponed until the end of pandemic. There is no data in the literature about the effect of a new epidemiological situation on bariatric patients. Methods: The study was designed as an online survey containing multiple open questions about bariatric care during the COVID-19 pandemic. The survey was conducted among pre- and postoperative bariatric patients. Results: Out of 800 respondents, 74.53% felt anxiety about their health in regard to the present epidemiologic state. Some (72.25%) were aware of the fact that obesity was an important risk factor that could impair the course of the COVID-19 disease. Almost 30% of respondents admitted having put on weight, significantly more in the group of preoperative patients (43.8% vs. 22.69%; *p* < 0.001). Only 20.92% of patients had a possibility of continuing direct bariatric care; 67.3% of patients had an opportunity of remote contact with a bariatric specialist, including online consultations, teleconsultations and social media meetings. Conclusions: Limited access to medical care and quarantine lockdown may result in a deterioration of long-time operation outcomes and lower weight losses. Patients should be encouraged to profit from online consultations with specialists and telemedicine to reduce the negative effects of the pandemic on their health.

## 1. Introduction

Obesity has reached epidemic proportions worldwide, and all evidence suggests that the situation is likely to get worse [1]. It is estimated that 65% of the adult population in the USA is overweight or obese [2]. Bariatric surgery is a mainstay treatment of obesity [3]. It is essential that patients receive long-term follow-up and monitoring to help them achieve the estimated weight loss, reduction of comorbidities and to prevent long-term problems that may arise following surgery [4].

A new disease appeared in the last quarter of the year 2019, causing a wide range of symptoms, from mild influenza-like illness to severe, life-threatening pneumonia. The infectious agent was found to be severe acute respiratory syndrome coronavirus 2 (SARS-CoV-2). In February 2020, the disease was designated by the World Health Organization (WHO) COVID-19, which stands for Coronavirus Disease 2019. On 11th March 2020, WHO declared COVID-19 a pandemic [5,6]. Until 22th April 2020, more than 2.61 million cases were reported across 185 countries, resulting in more than 182,000 deaths. In the same time, there were more than 10,000 COVID-19 cases reported and 400 people died because of COVID-19 pneumonia, but this data is most likely underestimated.

The new epidemiological situation has perturbed the functioning of healthcare systems around the world and led to changes in elective surgical care. It has not been confirmed yet that there is an increased incidence of COVID-19 pneumonia in obese patients. However, it is known that the process of treatment is less effective in people with comorbidities such as hypertension and diabetes mellitus, which are common in obese patients [7,8]. The International Federation for the Surgery of Obesity and Metabolic Disorders (IFSO) recommended that all elective metabolic and bariatric procedures, both surgical and endoscopic, should be postponed until the end of the pandemic [9]. The delay of operation may affect patients’ health in different ways, regardless if in the case of oncological or bariatric patients. While some data have already been gathered on the condition of surgery in a time of pandemic [10,11,12], there is hardly any data specifically about bariatric surgery and practically none about the effects of the COVID-19 pandemic on bariatric patients’ wellbeing.

The aim of the study was to investigate the impact of the COVID-19 pandemic on bariatric care from the patients’ point of view.

## 2. Methods

This study was designed as an online survey with the aim to collect data about bariatric care during the COVID-19 pandemic from patients in the course of qualification for bariatric surgery and patients after bariatric surgery. Survey contains 46 (multiple choice, open and Likert scale) questions. The questionnaire was evaluated and approved of by several independent experts in the field of bariatric surgery. The online survey was published and distributed via social media in cooperation with the Polish bariatric patients’ society, which integrates more than 1500 bariatric patients. The survey started on 9th April 2020 and was open until 17th April 2020. It was divided into four chapters: general information about the patient, life during the COVID-19 pandemic, bariatric care during the COVID-19 pandemic and life after the COVID-19 pandemic. Survey is shown in Appendix A. The project was supported by the Metabolic and Bariatric Chapter of Polish Surgeons’ Association (SCMiB). The data was completely anonymized and contained no patient identification data.

### 2.1. Statistical Analysis

Results are presented as means with standard deviation or medians with interquartile range. We performed the statistical analysis using StatSoft Statistica version 6.1 PL (StatSoft Inc., Tulsa, OK, USA). Normality of the data was tested with Shapiro–Wilk test. Continuous variables were compared with the Student’s *t*-test for normally distributed or Mann–Whitney U test for non-normally distributed data. Categorical variables were compared using the chi-square or Fisher test. Statistical significance was set at *p* < 0.05.

### 2.2. Ethical Considerations

The study was anonymous, performed in accordance with the ethical standards laid down in the 1964 Declaration of Helsinki and its latter amendments (Fortaleza). Participants were informed about the aim of the study, and informed consent was obtained electronically prior to the beginning of the survey. The study was approved by the Bioethics Committee of Jagiellonian University (1072.6120.103.2020).

## 3. Results

### 3.1. Basic Characteristics of the Patients

There were 800 participants, with the median age 39 (33–45) and body mass index (BMI) 34.26 (29.05–40.81) and mostly female (88%). The basic characteristics of the cases and the incidences of comorbidities are shown in Table 1. IQR: interquartile range. n/a: not applicable.

### 3.2. Life During the COVID-19 Pandemic

Only 6.64% of respondents had contact with patients with confirmed COVID-19 or were staying in quarantine. Some (21.9%) patients were treated in bariatric centers that currently manage COVID-19 patients. The majority (74.53%) of patients felt more anxiety/fear about their health in regard to the present epidemiologic state. Many (72.25%) were aware of the fact that obesity was an important risk factor that could impair the course of COVID-19. More than one-third of patients changed their eating habits during the epidemic, significantly less often after bariatric surgery. More than nine in ten patients did not increase physical activity. Half of patients did not gain weight, but almost 30% of respondents admitted to having put on weight, significantly more in the group of preoperative patients (43.8% vs. 22.69%; *p* < 0.001).

### 3.3. Bariatric Care During the COVID-19 Pandemic

Only 20.92% of patients had a possibility of continuing direct bariatric care during the COVID-19 pandemic, significantly less often in the group of preoperative patients (10.2% vs. 26.09%; *p* < 0.001). In 172 cases (69.36%), the date of bariatric surgery was postponed due to the COVID-19 pandemic; in 3.63% cases, it was the patient’s decision; in 65.73% cases, it was the decision of the bariatric center; in 30.65% cases, the date of the surgery did not change. In the present situation, 50.33% of respondents decided to undergo bariatric surgery in spite of the pandemic, considerably more likely in the preoperative group (67.72% vs. 41.62%; *p* < 0.001). Some (60.67%) patients, both from the preoperative and postoperative groups, had their control visits postponed by the bariatric centers. A number (67.3%) of patients had an opportunity of remote contact with a bariatric specialist, including online consultations, teleconsultations and social media meetings. Regardless of the risk of becoming infected with COVID-19, 42.69% of patients would like to have a visit in a bariatric clinic, for the most part in the preoperative group (57.59% vs. 35.47%; *p* < 0.001). Most patients affirmed the necessity of the continuous support of bariatric surgeons, dietician nutritionists and psychologists. The vast majority of patients accept and are satisfied with teleconsultations as a form of contact with a specialist or qualification for bariatric treatment. More than 60% of patients did not have the possibility of doing diagnostic tests related to bariatric care, and more than 90% had problems with their availability. Almost 20% of patients admitted to having anxiety about health problems that might have resulted from the limited access to bariatric care, mostly in the postoperative group (43.61% vs. 29.07%; *p* < 0.001).

### 3.4. Life after the COVID-19 Pandemic

An important question that was part of the last chapter of the survey was when the bariatric procedures should be restarted. Preoperative patients compared with postoperative patients significantly more often declared that bariatric procedures should be resumed as soon as the daily number of COVID-19 infections would start to decrease (51.75% vs. 15.9%). Other possible answers were: as soon as the WHO would declare the end of the pandemic (30.74% of the preoperative group vs. 54.93% of the postoperative group), the discharge from the hospital of the last COVID-19 patient (13.23% vs. 14.89%) and after the introduction of a COVID-19 vaccine (4.28% vs. 14.29%).

Some (47.14%) patients recognized the priority in treating patients with cancer before bariatric patients. A number (67.93%) of patients stated that patients whose operations were postponed due to the COVID-19 pandemic should be treated first when the bariatric procedures are resumed. A number (52.80%) of patients accepted the possibility of a requalification for bariatric treatment and repeated diagnostic tests after the pandemic.

The majority of patients still wanted to undergo surgery (88.01%) after the pandemic and did not consider changing their bariatric center (87.59%). Only 2.55% of patients were thinking about changing the type of bariatric procedure planned.

The majority of patients (93.99%) planned to increase their physical activity after the pandemic, more often in the preoperative group (97.29% vs. 92.37%; *p* = 0.006). Some (63.6%) patients considered changing their eating habits after the pandemic, significantly more often in the preoperative group (85.77% vs. 52.79%; *p* < 0.001). Detailed data is presented in Table 2.

## 4. Discussion

Our study based on a national range survey among pre- and postoperative bariatric patients presents the impact of the COVID-19 pandemic on the life of bariatric patients. The novelty of our study was the analysis of the impact of the COVID-19 pandemic on bariatric care from the patients’ point of view. The fact of postponing elective bariatric surgery procedures has affected the lives of many patients waiting for the operation. The quarantine lockdown has influenced lifestyles and dietary regimens of pre- and postoperative patients. Although the vast majority of responders did not have contact with COVID-19 infected patients, most patients felt anxious about their health in regards to the present epidemiologic state. The majority of responders were aware of the fact that obesity was an important risk factor that could impair the course of COVID-19 disease. More than two-thirds of preoperative patients had their operation postponed and, more than a half, their control visits. Almost 70% of patients had a possibility of online consultations with a specialist and the use of telemedicine. Most patients before surgery wanted to undergo surgical treatment after the pandemic. Only less than half the patients recognized the priority of treating oncological patients; the others preferred a simultaneous restart of all kinds of surgical procedures. The majority of patients planned on increasing their physical activity and changing eating habits after the pandemic.

The COVID-19 pandemic has a tremendous impact on the daily routine and quality of life of billions of people worldwide. Self-isolation and quarantine lockdown cause additional distress and increase the levels of fear and anxiety [13]. Bariatric patients are in a high-risk group of increased eating psychopathology and trouble in self-management in such a situation of emotional distress. Followed by a reduction of physical activity due to lockdown, possible financial difficulties and trouble with food availability, the pandemic may result in difficulties with optimum weight losses, possible weight gains and the deterioration of long-term outcomes [14].

The importance of a multidisciplinary team in bariatric care has been well-established [15,16]. The success of an operation is mostly determined by postoperative care, and patients must remain in regular contact not only with their bariatric surgeon but, also, dietitian, psychologist and a specialist in internal medicine or endocrinologist [4,17]. Our study showed that the present state of the pandemic is a major obstacle for the patients with maintaining contact and getting help from their bariatric team. As patients have limited access to ambulatory clinics, new ways of communication have had to be quickly developed. Before the era of pandemics, telemedicine was used in our country only in very limited situations, mostly for teleconferences between specialists and the live consulting of test results. Due to the lockdown, it had to develop quickly as the most important tool of present communication between patients, doctors, dietitians and psychologists. Telemedicine and remote consultations are proven to be effective in fighting distress and reducing the level of psychological disorders in bariatric patients [14]. According to the data from the Central Statistical Office from 2019, 86.7% of Polish households had internet access. Therefore, the general majority of patients after bariatric procedures have internet access, and the results of the study should not be influenced by the problem of internet and telemedicine availability [18].

There have been no surveys about COVID-19 conducted among bariatric patients yet, so we can compare our study to only similar studies in other fields, though they are also scarce at this time. Wolf et al. conducted a survey in a group of 630 patients with at least one chronic disease [19]. Only 24.6% patients were “very worried” about getting COVID-19, and 12.95% of patients were “not worried at all”. More than half the patients (58.6%) admitted that coronavirus had a high impact on their daily routine, and only 20.8% of respondents felt “very prepared” for the outbreak. The study by Wolf et al. revealed profound gaps in the patients’ knowledge and level of concern about the virus. In our study, most patients (more than 74%) were worried about the risk brought by the COVID-19 pandemic. Another survey-based report regarding patients’ awareness, attitudes and actions related to COVID-19 was published about people living with HIV in China [20]. The majority of the respondents felt well-informed; they were concerned about specific protective measures, and 64.15% reported difficulties in accessing antiretroviral medicines due to lockdown. Almost 30% of respondents declared a need for sociopsychological support. There also was a report published basing on a survey among Indian ophthalmologists regarding the effects of the pandemic on their practice and patient care, 77.5% of whom decided to use different forms of telemedicine [21].

### Limitations of the Study

The possible limitations of our study can be the recall bias and the subjectivity of patients’ opinions. Another limitation was that the survey was conducted only among Polish bariatric patients who were able to fill it out by means of the internet. All respondents were voluntary members of the bariatric patient support group, which introduced them to the purpose and methodology of the study. Moreover, there was no incentive to introduce dishonesty into the responses. However, direct control of the respondents was currently not possible due to the ongoing pandemic, and it is unfortunately a limitation of the study. Additionally, in order to obtain the highest possible number of responders in a considerably short period of time, we decided to post the questionnaire on the Polish bariatric patients’ society website, and we were not able to calculate the response rate.

## 5. Conclusions

The COVID-19 pandemic affected the functioning of Polish bariatric surgery, as elective procedures were postponed until the end of the pandemic. Patients have problems with access to bariatric surgeons, dieticians, nutritionists and psychologists, who together form teams taking care of bariatric patients. Limited access to medical care and quarantine lockdown may result in patients’ bad eating habits, lack of physical exercise and psychological distress and lead to the deterioration of long-time operation outcomes and lower weight losses. Patients should be encouraged to profit from online consultations with specialists and telemedicine to reduce the negative effects of the pandemic on their health.

## Figures and Tables

**Table 1:** Basic characteristics.

	All	Preoperative Patients	Postoperative Patients	*p*-Value
*n* (%)	800 (100%)	258 (32%)	542 (68%)	n/a
Median age, years (IQR)	39 (33–45)	37 (32–43)	39 (33–46)	0.005
Males/Females, *n* (%)	97/703 (12%/88%)	33/225 (12%/88%)	64/478 (12%/88%)	0.647
Median BMI, kg/m^2^ (IQR)	34.26 (29.05–40.81)	42.24 (38.64–47.75)	31.18 (27.36–35.43)	<0.001
Insulin resistance, *n* (%)	224 (28%)	86 (33.33%)	138 (25.46%)	0.020
Type 2 diabetes mellitus, *n* (%)	93 (11.63%)	31 (12.02%)	62 (11.44%)	0.812
Obstructive sleep apnea, *n* (%)	63 (7.88%)	23 (8.91%)	40 (7.38%)	0.451
Arterial hypertension, *n* (%)	265 (33.13%)	86 (33.33%)	179 (33.03%)	0.931
Dyslipidemia, *n* (%)	68 (8.5%)	27 (10.47%)	41 (7.56%)	0.169
Arthritis/Joint pain, *n* (%)	272 (34%)	106 (41.09%)	166 (30.63%)	0.003

**Table 2:** Results of the questionnaire.

Questions	Answers	All	Preoperative Patients	Postoperative Patients	*p*-Value
**Life During the COVID-19 Pandemic**					
Have any of your relatives or friends currently contracted COVID-19 or are in quarantine?	Yes	53 (6.64%)	8 (3.1%)	45 (8.33%)	0.021
	No	721 (90.35%)	242 (93.80%)	479 (88.70%)	
	I do not know	24 (3.01%)	8 (3.1%)	16 (2.96%)	
Do you feel more anxiety/fear about your health/life in regards to the current epidemiologic state?	Yes	594 (74.53%)	201 (78.21%)	393 (72.78%)	0.099
Are you aware of the fact that obesity is important risk factor impairing the course of infection of COVID-19?	Yes	578 (72.25%)	195 (75.58%)	383 (70.66%)	0.147
Did you change eating habits due to the epidemy?	Yes	274 (34.29%)	120 (46.51%)	154 (28.47%)	<0.001
Has your physical activity changed due to the limited possibilities of going outside, closing places of recreation and sports facilities?	Yes—increased	63 (7.88%)	22 (8.53%)	41 (7.56%)	0.789
	Yes—decreased	481 (60.13%)	151 (58.53%)	330 (60.89%)	
	No	256 (32%)	85 (32.95%)	171 (31.55%)	
Are you exercising at home by your own?	Yes	582 (72.75%)	173 (67.05%)	409 (75.46%)	0.013
How has the pandemic influenced your body weight?	Increase	236 (29.5%)	113 (43.8%)	123 (22.69%)	<0.001
	Decrease	154 (19.25%)	24 (9.3%)	130 (23.99%)	
	No changes	410 (51.25%)	121 (46.9%)	289 (53.32%)	
Bariatric Care During the COVID-19 Pandemic					
Do you currently have the option of continuing bariatric treatment?	Yes	164 (20.92%)	26 (10.20%)	138 (26.09%)	<0.001
Has the date of bariatric surgery been postponed due to the COVID-19 pandemic?	Yes—my own decision	15 (2.68%)	9 (3.63%)	6 (1.93%)	<0.001
	Yes—decision of the hospital administration	188 (33.63%)	163 (65.73%)	25 (8.04%)	
	No	356 (63.69%)	76 (30.65%)	280 (90.03%)	
In spite of the pandemic and the associated risk of developing COVID-19, would you undergo bariatric surgery in the current situation?	Yes	383 (50.33%)	172 (67.72%)	211 (41.62%)	<0.001
Has the date of the visit to the surgery center been moved due to the COVID-19 pandemic?	Yes—my own decision	45 (6.86%)	14 (5.58%)	31 (7.65%)	<0.001
	Yes—decision of the hospital administration	353 (53.81%)	166 (66.14%)	187 (46.17%)	
	No	258 (39.33%)	71 (28.29%)	187 (46.17%)	
Do you have the opportunity to contact doctors providing bariatric treatment, e.g., online consultations, teleconsultations and social media?	Yes	529 (67.30%)	149 (58.2%)	380 (71.7%)	<0.001
In spite of the pandemic and the associated risk of developing COVID-19, would you visit a bariatric clinic in the current situation?	Yes	336 (42.69%)	148 (57.59%)	188 (35.47%)	<0.001
How do you assess the safety of meetings in a bariatric clinic in terms of the possibility of developing COVID-19?	Median score (IQR)	5 (3-8)	5 (2-7)	6 (4-8)	<0.001
Do you think that remote advice for bariatric patients during a pandemic is needed?	Median score (IQR)	10 (8-10)	10 (8-10)	10 (8-10)	0.216
Do you think that remote advice of bariatric surgeons for bariatric patients during a pandemic is needed?	Median score (IQR)	9 (7-10)	9 (7-10)	9 (7-10)	0.438
Do you think that remote advice of dieticians for bariatric patients during a pandemic is needed?	Median score (IQR)	10 (8-10)	10 (8-10)	10 (8-10)	0.036
Do you think that remote advice of psychologists for bariatric patients during a pandemic is needed?	Median score (IQR)	10 (8-10)	10 (8-10)	10 (8-10)	0.016
Have you used online support groups during a pandemic?	Yes	422 (53.28%)	140 (54.69%)	282 (52.61%)	0.584
I consider the participation of support groups and patient organizations during a pandemic to be:	Median score (IQR)	9 (7-10)	9 (7-10)	8 (6-10)	0.016
Do you accept teleconsultations as a form of treatment or qualification for bariatric treatment?	Median score (IQR)	9 (6-10)	9 (6-10)	10 (6-10)	0.082
How satisfied are you with teleconsultations?	Median score (IQR)	8 (5-10)	8 (5-10)	7 (5-10)	0.018
Do you have the opportunity to perform the tests recommended by the attending physician?	Yes	69 (8.96%)	19 (7.45%)	49 (9.72%)	0.560
	Yes—but limited	232 (30.57%)	81 (31.76%)	151 (29.96%)	
	No	459 (60.47%)	155 (60.78%)	304 (60.32%)	
Has the situation of limited access to bariatric care caused any health problems for you?	Yes	133 (17.05%)	75 (29.07%)	58 (43.61%)	<0.001
Life after the COVID-19 Pandemic					
After the pandemic, will you still want to undergo surgery?	Yes	499 (88.01%)	258 (100%)	241 (77.99%)	n/a
Do you intend to undergo surgery at the same unit?	Yes	494 (87.59%)	254 (98.45%)	240 (78.43%)	<0.001
Have you changed your decision about the type of surgery after the pandemic?	Yes	20 (2.55%)	3 (1.17%)	17 (5.52%)	0.005
At what point should bariatric procedures resume?	As soon as the daily number of COVID-19 infections start to decrease	212 (28.12%)	133 (51.75%)	79 (15.90%)	<0.001
	After the introduction of a COVID-19 vaccine	82 (10.88%)	11 (4.28%)	71 (14.29%)	
	As soon as the WHO will declare the end of the pandemic	352 (46.68%)	79 (30.74%)	273 (54.93%)	
	After discharge of the last COVID-19 patient from the hospital	108 (14.32%)	34 (13.23%)	74 (14.89%)	
After what time should bariatric procedures be resumed?	After the waiting list for oncologic procedures will be shortened	363 (47.14%)	82 (31.78%)	281 (54.88%)	<0.001
	At the same time of the oncologic procedures	122 (15.84%)	62 (24.03%)	60 (11.72%)	
	Due to a low risk before the oncologic procedures	75 (9.74%)	47 (18.22%)	28 (5.47%)	
	No opinion	210 (27.27%)	67 (25.97%)	143 (27.93%)	
Are you ready to undergo a requalification and examination cycle due to postponed surgery?	Yes	349 (52.80%)	174 (67.44%)	175 (43.42%)	<0.001
	No	86 (13.01%)	54 (20.93%)	32 (7.94%)	
	No opinion	226 (34.19%)	30 (11.63%)	196 (48.64%)	
Do you consider it necessary to postpone the dates of new qualifications and bariatric surgeries in the period after the pandemic ends so that postponed patients due to a pandemic could be treated first?	Yes	494 (67.39%)	168 (65.37%)	326 (68.49%)	<0.001
	No	76 (10.37%)	47 (18.29%)	29 (6.09%)	
	No opinion	163 (22.24%)	42 (16.34%)	121 (25.42%)	
Do you have a plan to increase physical activity after the pandemic?	Yes	735 (93.99%)	251 (97.29%)	484 (92.37%)	0.006
Do you have a plan to change your eating habits after the pandemic?	Yes	491 (63.60%)	217 (85.77%)	274 (52.79%)	<0.001

## Appendix A. Questionnaire for Bariatric Patients

**Questions**	**Answers**
**Basic Characteristics**	
Age	(Number)
Sex	Male/Female
Weight	(Number)
Height	(Number)
Co-morbidities	Insulin resistance
	Type 2 diabetes mellitus
	Obstructive sleep apnea
	Arterial hypertension
	Dyslipidemia
	Arthritis/Joint pain
What is your bariatric status	Pre-operative
	Post-operative
**Life During COVID-19 Pandemic**	
Do any of your relatives or friends is currently contracted with COVID-19 or in quarantine?	Yes
	No
	I do not know
Do you feel more anxiety/fear about your health/life in regards to current epidemiologic state?	Yes
	No
Are you aware of the fact that obesity is important risk factor impairing the course of infection of COVID-19?	Yes
	No
Did you changed eating habits due to the epidemy?	Yes
	No
Has your physical activity changed due to the limited possibilities of going outside, closing places of recreation and sports facilities?	Yes—increased
	Yes—decreased
	No
Are you exercising at home by your own?	Yes
	No
How the pandemic influenced your body weight?	Increase
	Decrease
	No changes
**Bariatric Care During COVID-19 Pandemic**	
Do you currently have the option of continuing bariatric treatment?	Yes
	No
Has the date of bariatric surgery been postponed due to the COVID-19 pandemic?	Yes—my own decision
	Yes—decision of the hospital administration
	No
In spite of the pandemic and the associated risk of developing COVID-19, would you undergo bariatric surgery in the current situation?	Yes
	No
Has the date of the visit to the surgery center been moved due to the COVID-19 pandemic?	Yes—my own decision
	Yes—decision of the hospital administration
	No
Do you have the opportunity to contact doctors providing bariatric treatment, e.g., online consultations, tele-consultations, social media?	Yes
	No
In spite of the pandemic and the associated risk of developing COVID-19, would you visit a Bariatric Clinic in the current situation?	Yes
	No
How do you assess the safety of meetings in the Bariatric Clinic in terms of the possibility of developing COVID-19?	From 1 to 10
Do you think that remote advice for bariatric patients during a pandemic is needed?	From 1 to 10
Do you think that remote advice of bariatric surgeon for bariatric patients during a pandemic is needed?	From 1 to 10
Do you think that remote advice of dietician for bariatric patients during a pandemic is needed?	From 1 to 10
Do you think that remote advice of psychologist for bariatric patients during a pandemic is needed?	From 1 to 10
Have you used online support groups during a pandemic?	Yes
	No
I consider the participation of support groups and patient organizations during a pandemic to be:	From 1 to 10
Do you accept tele-consultations as a form of treatment or qualification for bariatric treatment?	From 1 to 10
How satisfied are you with tele-consultations?	From 1 to 10
Do you have the opportunity to perform the tests recommended by the attending physician?	Yes
	Yes—but limited
	No
Has the situation of limited access to bariatric care caused any health problems to you?	Yes
	No
**Life after COVID-19 Pandemic**	
After the pandemic, will you still want to undergo surgery?	Yes
	No
Do you intend to undergo surgery in the same unit?	Yes
	No
Have you changed your decision about the type of surgery after the pandemic?	Yes
	No
At what point should bariatric procedures resume?	As soon as daily number of COVID-19 infections start to decrease
	After introduction of COVID-19 vaccine
	As soon as WHO will declare end of pandemic
	After discharge of the last COVID-19 patient from hospital
After what time should bariatric procedures be resumed?	After the waiting list for oncologic procedures will be shortened
	At the same time of oncologic procedures
	Due to low risk before oncologic procedures
	No opinion
Are you ready to undergo a re-qualification and examination cycle due to postponed surgery?	Yes
	No
	No opinion
Do you consider it necessary to postpone the dates of new qualifications and bariatric surgeries in the period after the pandemic ends so that postponed patients due to a pandemic could be treated first?	Yes
	No
	No opinion
Do you have a plan to increase physical activity after the pandemic?	Yes
	No
Do you have a plan to change your eating habits after the pandemic?	Yes
	No
